# Guava leaf extract suppresses osteoarthritis progression in a rat anterior cruciate ligament transection model

**DOI:** 10.1002/fsn3.601

**Published:** 2018-03-10

**Authors:** Keiko Kawasaki, Takashi Fushimi, Junji Nakamura, Noriyasu Ota

**Affiliations:** ^1^ Biological Science Research Laboratories Kao Corporation Tochigi Japan; ^2^ Kansei Science Research Laboratories Kao Corporation Tochigi Japan

**Keywords:** ADAMTS‐5, cartilage destruction, ellagic acid, guava leaf extract, osteoarthritis

## Abstract

Guava leaf extract and ellagic acid, one of its polyphenolic components, inhibit the activity of a disintegrin and metalloproteinase with thrombospondin type 5 (ADAMTS‐5), which is associated with aggrecan degeneration during the early stage of osteoarthritis (OA). To investigate the efficacy of guava leaf extract for preventing OA, we examined the effect of its dietary intake on cartilage destruction in anterior cruciate ligament‐transected (ACLT) rats. Rats were randomly assigned to four groups: ACLT control rats fed with control diet, ACLT rats fed with diet containing 0.2% guava leaf extract, ACLT rats fed with diet containing 0.5% guava leaf extract, and sham‐operated rats fed with control diet. Mankin's scores, an index of cartilage damage, were higher in rats that underwent ACLT. Guava leaf extract treatment dose‐dependently led to lower Mankin's scores and higher concentrations of ellagic acid in the serum and synovial membrane. Ellagic acid levels in the synovial membrane negatively correlated with cartilage destruction scores. These results suggest that dietary guava leaf extract suppresses OA progression in ACLT rats through ellagic acid‐mediated inhibition of early joint destruction.

## 1 INTRODUCTION

Japan is currently facing a “super‐aged” society, with disability‐free life expectancy of the population estimated to be ten years shorter than the average life expectancy for both sexes (Nakamura, 2009). Thus, prolonging the disability‐free life expectancy will help the elderly lead more enriched lives.

Joint diseases are often precursors to physical disabilities. Osteoarthritis (OA), the most common type of joint disease, is prevalent in developed countries, especially among the elderly. As the proportion of senior citizens in the population increases, the number of patients suffering from OA is also expected to increase. In Japan, OA affects more than 25 million people and is a major reason the elderly require nursing care (Yoshimura et al., 2009). Thus, effective treatments against OA are essential. Various symptomatic treatments have been proposed to alleviate OA, including knee replacement arthroplasty, arthroscopic surgery, intra‐articular injection of hyaluronic acid or steroids, and pharmacological therapies. However, neither preventive measures nor primary cures for OA have been established. Hence, alternative methods to address OA, such as the consumption of functional foods, are becoming more popular.

Aggrecanase‐mediated degradation of aggrecan is a significant event in the early stage of OA. A disintegrin and metalloproteinase with thrombospondin type 5 (ADAMTS‐5) plays an important role in this process (Glasson et al., 2005; Stanton et al., 2005; Verma & Dalal, 2011). Thus, inhibition of ADAMTS‐5 is one way of preventing OA. Since the discovery of an ADAMTS‐5 inhibitor was initially reported (Yao et al., 2001), several new inhibitors have been proposed (Shiozaki et al., 2011). However, no significant progress has been achieved in the development of daily functional foods or therapeutic medicines to address OA.

In our preliminary experiments, we discovered that guava leaf extract among natural plant extracts strongly suppresses ADAMTS‐5 activity (data not shown). Guava has been reported to contain various polyphenols including ellagic acid (Anand, Kumar, Kumar, & Hedina, 2016; Arshiya, 2013; Lin & Yin, 2012), which has strong ADAMTS‐5 inhibitory activity. Guava, belonging to the family Myrtaceae, is an evergreen tree that grows in torrid and subtropical zones. Its leaves, roots, and fruits have been used in folk medicine owing to their antibacterial, antihyperglycemia, and anti‐inflammatory properties (Yan, Lee, Kong, & Zhang, 2013). Additionally, guava leaves have been used to prepare tea in those regions, and it has been used as a functional food in Japan. Although guava is commonly consumed in these areas, very little is known about the effects of dietary guava on OA.

In this study, we demonstrate the suppressive effects of guava leaf extract on OA using a rat model of anterior cruciate ligament transection (ACLT).

## 2 MATERIALS AND METHODS

### 2.1 Guava leaf extract

Guava leaf extract was purchased from Matsuura Yakugyo Co. (Aichi, Japan). It contained 2.0% ellagic acid and other polyphenols (1.0% quercetins, 0.2% catechins), and its composition was as follows: 70.6% carbohydrate, 1.1% fat, 2.9% protein, 7.6% fiber, 14.0% ash, and 3.8% water. In preliminary experiments, we evaluated the ADAMTS‐5 inhibitory activity of guava leaf extract by measuring aggrecan fragments produced by ADAMTS‐5 in a reaction mixture containing the substrate, enzyme, and test sample (Karai et al., 2012). The inhibitory activity of guava leaf extract was more than ten times higher, and ellagic acid was more than three times higher than that of actinonin, the positive control.

### 2.2 Animals and treatment protocol

Animal experimental protocols were approved by the Ethics Committee of Kao Corporation. Male Sprague Dawley rats (8 weeks old) were obtained from Japan SLC (Shizuoka, Japan). Rats were assigned to four experimental groups as follows: ACLT rats fed a control diet (ACLT/Cont), ACLT rats fed a diet containing low‐dose (0.2%) guava leaf extract (ACLT/LG), ACLT rats fed a diet containing high‐dose (0.5%) guava leaf extract (ACLT/HG), and sham‐operated rats fed a control diet (sham). Rats were acclimated for 1 week, and then, the anterior cruciate ligaments from both legs (excluding rats in the sham group) were transected as previously described (Kamekura et al., 2005) under isoflurane‐oxygen anesthesia. Rats in the sham group underwent arthrotomy without ACLT in both legs. Guava tea is typically extracted from several grams of guava leaf, and Japanese adult ingests approximately 2,000 g of food per day on average. Therefore, the dosage in this study was set to 0.2% and 0.5% within the ingestion level of daily life.

The control, LG, and HG groups were fed an AIN‐93 powder diet containing 0, 0.2%, and 0.5% guava leaf extract in place of starch, respectively. Rats were given 20‐g experimental diet/day after surgical treatment for 10 weeks. They had free access to water and were housed in a temperature‐ and humidity‐controlled chamber with a 12‐hr light‐dark cycle (lights‐on time: 7:00 a.m. – 7:00 p.m.). Body weight was recorded weekly, and serum samples were taken at 0, 1, and 10 weeks postsurgery. Both knee joints containing the femurs, tibias, and synovial membranes were removed following euthanasia at 10 weeks postsurgery.

### 2.3 Histological analysis

Histological analysis was performed as previously described (Galois et al., 2004; Mankin, Dorfman, Lippiello, & Zarins, 1971). Briefly, the right knee joint was dissected, fixed in 10% neutral‐buffered formalin, and decalcified with 10% ethylenediaminetetraacetic acid (EDTA). After dehydration with increasing concentrations of ethanol and embedding in paraffin, serial frontal sections of the ACL (4–5 μm thick) were prepared for histological observation. The sections were stained with hematoxylin–eosin and safranin O to observe OA development. The severity of OA was evaluated using a scale adapted from the Mankin's scoring method by two independent observers who were blinded to the experimental groups. The scores from the two observers were averaged. The scoring categories consisted of cartilage destruction (score: 0–6), chondrocyte cellularity (score: 0–3), loss of proteoglycans (score: 0–4), and tidemark change (score: 0–2). Scoring was assessed for four parts, including the inner and outer tibias and the inner and outer femurs. The final score was the combined score of all four parts (final score range: 0–60).

### 2.4 Analysis of serum and synovial membrane

#### 2.4.1 Ellagic acid

Ellagic acid concentrations were determined as previously reported (Espin et al., 2007) with minor modifications. Briefly, serum samples (300 μl) were mixed with an antioxidant solution (111 μl) containing 0.2 g/ml ascorbic acid and 1 mg/ml EDTA and o‐phosphoric acid (6 μl) to break the protein–phenol bond. After vortexing for 2 min, 1.5 ml acetonitrile was added; the mixture was further vortexed for 2 min and sonicated for 1 min. The mixture was then centrifuged at 14,000 *g* for 10 min at 4°C. The supernatant was collected and dried under a nitrogen purge. The residue was reconstituted with a methanol/hydrochloric acid (99.9:0.1) solution (100 μl); a 20‐μl volume sample was applied to an HPLC system.

Synovial membranes (approximately 10 mg) were homogenized in a methanol/hydrochloric acid/water (79.9:0.1:20) solution (200 μl) before mixing with an antioxidant solution (111 μl) containing 0.2 g/ml ascorbic acid and 1 mg/ml EDTA and o‐phosphoric acid (4 μl). Subsequent procedures were the same as described above.

A Waters LC system (Milford, MA, USA) equipped with Empower software, a diode array detector, and an XBridge C18 3.5‐μm octadecylsilyl (ODS) column (4.6 mm × 150 mm; Waters) were used for ellagic acid analysis. A binary pump was connected to eluents A (0.1% trifluoroacetic acid; TFA) and B (0.1% TFA and 99.9% acetonitrile). The mobile phase was programmed as follows: an isocratic elution of 88% A for 15 min, a stepwise gradient down to 5% A for 10 min to wash column, followed by a stepwise gradient to 88% A for 15 min to equilibrate column. The column temperature was maintained at 40°C, and the flow rate was set to 1 ml/min. Serum ellagic acid was analyzed by HPLC‐MS/MS, and synovial membrane ellagic acid was detected at 254 nm.

Ellagic acid concentrations in the serum and synovial membranes were only measured in rats that underwent ACLT, as ellagic acid was not detected in normal rats during preliminary experiments.

#### 2.4.2 Cartilage oligomeric matrix protein (COMP)

Levels of serum COMP, a cartilage degradation marker, were measured using a commercially available enzyme‐linked immunosorbent assay (ELISA) kit (Animal COMP ELISA; AnaMar Medical AB, Lund, Sweden). Data were analyzed by defining the value at week 0 as baseline (100).

### 2.5 Statistical analysis

All experimental data were analyzed using SPSS (version 23.0; SPSS, Inc., Chicago, IL, USA) or SAS for nonparametric test (version 9.1; SAS Institute Inc., Cary, NC, USA). For intergroup comparisons, a Dunnett's multiple comparison parametric test or a Steel's multiple comparison nonparametric test was performed by setting the value for the ACLT/Cont group as the reference. When the normal distribution or the equal variance was not statistically assured, a nonparametric test was adopted. Differences in *p *<* *.05 were considered statistically significant. Data are presented as the means ± standard deviation (*SD*). Because the distribution of synovial membrane ellagic acid content was not normal, the relationship with the cartilage destruction score was analyzed by Spearman's correlation.

## 3 RESULTS

### 3.1 Body weight

During the test period, there were no significant differences in rat body weight among all groups (Table 1). In addition, no side effects were observed upon treatment.

**Table 1:** Change in body weight over time of sham‐operated and anterior cruciate ligament‐transected (ACLT) rats fed with diets containing 0% guava leaf extract (Sham and ACLT/Cont), 0.2% guava leaf extract (ACLT/LG), or 0.5% guava leaf extract (ACLT/HG)

Group	Acclimation period	Week 0	Week 1	Week 2	Week 3	Week 4	Week 5	Week 6	Week 7	Week 8	Week 9	Week 10
Sham	285 ± 12	292 ± 13	347 ± 11	400 ± 13	436 ± 17	466 ± 21	487 ± 27	518 ± 27	539 ± 28	555 ± 31	573 ± 35	596 ± 36
ACLT/Cont	286 ± 8	319 ± 33	343 ± 13	402 ± 18	444 ± 20	469 ± 23	487 ± 21	510 ± 22	533 ± 25	547 ± 26	564 ± 28	586 ± 22
ACLT/LG	287 ± 11	310 ± 15	342 ± 14	394 ± 17	434 ± 20	455 ± 22	470 ± 23	491 ± 25	509 ± 27	527 ± 28	545 ± 31	572 ± 34
ACLT/HG	286 ± 5	311 ± 10	347 ± 10	403 ± 13	442 ± 11	472 ± 15	489 ± 17	512 ± 16	536 ± 16	557 ± 19	577 ± 22	604 ± 23

Values represent the mean ± *SD* of seven rats per group.

### 3.2 Histological analysis

**Figure 1:**
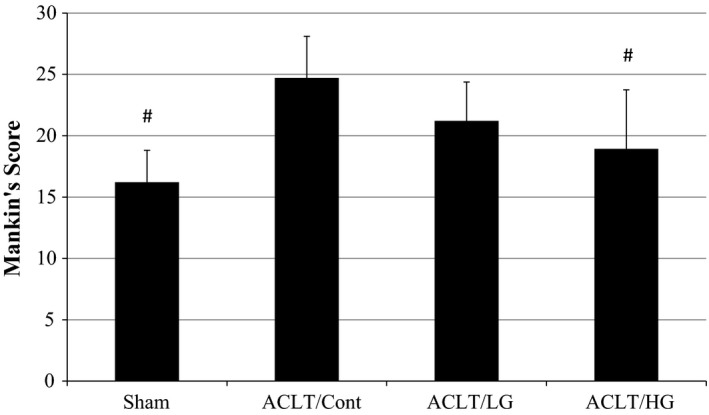
Mankin's score of the right knee joints of sham‐operated and anterior cruciate ligament‐transected (ACLT) rats fed diets containing 0% guava leaf extract (Sham and ACLT/Cont), 0.2% guava leaf extract (ACLT/LG), or 0.5% guava leaf extract (ACLT/HG). The knee joints were dissected on week 10. Values represent the means and *SD* (as shown as vertical bars). ^#^Mean value is significantly different from that of the ACLT/Cont group (*p* < .05, a Dunnett's multiple comparison test)

**Figure 2:**
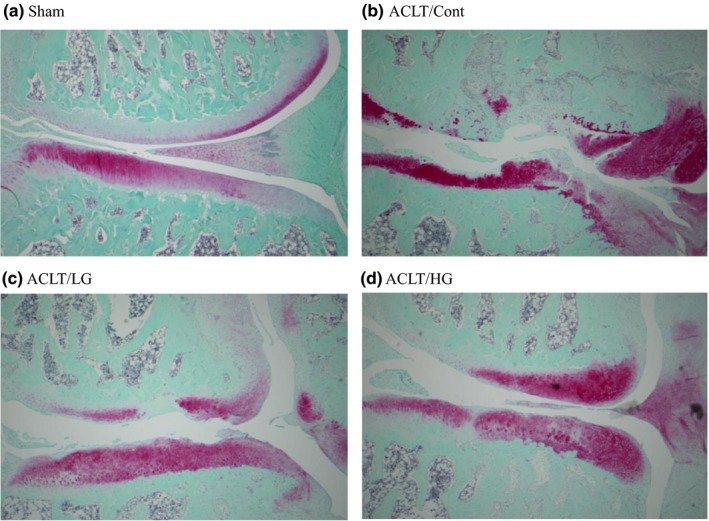
Histological pictures of the right inner knee joints of sham‐operated and anterior cruciate ligament‐transected (ACLT) rats fed diets containing 0% guava leaf extract (Sham: a and ACLT/Cont: b), 0.2% guava leaf extract (ACLT/LG: c), or 0.5% guava leaf extract (ACLT/HG: d). The knee joints were dissected on week 10. Cartilage stained purple with safranin O, whereas the white part was joint gap

Mankin's scores of the ACLT/Cont group were significantly higher than those of the Sham and ACLT/HG groups (Figure 1). Although the score of the ACLT/LG group was also lower than that of the ACLT/Cont group, the difference was not significant. Typical histological pictures of each group are shown in Figure 2. Cartilage stained purple with safranin O, whereas the white part indicates the joint gap. Knee joint states in ACLT/LG and ACLT/HG groups were cleaner than those in ACLT/Cont group. These observations suggest that treatment with guava leaf extract reduces knee joint destruction in a dose‐dependent manner.

### 3.3 Ellagic acid in the serum and synovial membrane

**Figure 3:**
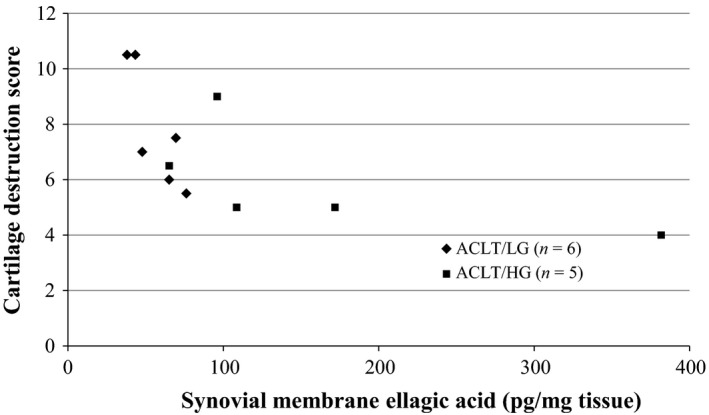
Plot of cartilage destruction score against synovial membrane ellagic acid concentration in anterior cruciate ligament‐transected (ACLT)/low guava (LG) and ACLT/high guava (HG) group rats fed with diets containing 0.2% and 0.5% guava leaf extract, respectively. The negative correlation between ellagic acid content and the cartilage destruction score was significant (Spearman's ρ = −0.804, *p* < .005)

The ACLT/LG and ACLT/HG groups exhibited higher ellagic acid concentrations in the serum and synovial membrane than those of the ACLT/Cont group (Table 2). The increase in ellagic acid concentrations directly correlated with higher doses of guava leaf extract. Although ellagic acid concentrations in the synovial membrane did not significantly correlate with the overall Mankin's score, they did parallel the score for cartilage destruction, which is one component of the Mankin's score (Figure 3).

**Table 2:** Serum and synovial membrane ellagic acid concentration in anterior cruciate ligament‐transected (ACLT) rats fed with diets containing 0% guava leaf extract (ACLT/Cont), 0.2% guava leaf extract (ACLT/LG), or 0.5% guava leaf extract (ACLT/HG)

	ACLT/Cont	ACLT/LG	ACLT/HG
Serum (ng/ml)	2.20 ± 4.92 (*n* = 5)	6.00 ± 5.48 (*n* = 5)	8.60 ± 4.83 (*n* = 5)
Synovial membrane (pg/mg tissue)	0.0 ± 0.0 (*n* = 5)^a^	56.7 ± 14.3 (*n* = 6)^b^	164.6 ± 114.0 (*n* = 5)^b^

Values represent the mean ± *SD* of five or six rats per group.

a In this group, ellagic acid was not detected.

b Mean value is significantly different from that of the ACLT/Cont group (*p* < .05, a Steel's multiple comparison test).

### 3.4 Serum COMP levels

Rats that underwent ACLT had higher serum COMP levels after 1 week than those of the sham group (Table 3). The ACLT/Cont group displayed significantly higher serum COMP levels than those of the sham group at weeks 1 and 10. However, there were no significant differences among the three ACLT groups at these time points.

**Table 3:** Change in serum cartilage oligometric matrix protein (COMP) level over time of sham‐operated and anterior cruciate ligament‐transected (ACLT) rats fed with diets containing 0% guava leaf extract (Sham and ACLT/Cont), 0.2% guava leaf extract (ACLT/LG), or 0.5% guava leaf extract (ACLT/HG). Serum COMP levels are calculated by defining the value at week 0 as baseline (100)

Group	Week 0	Week 1	Week 10
Sham		96.0 ± 13.0^a^	46.4 ± 3.4^a^
ACLT/Cont	100	126.1 ± 23.6	71.5 ± 6.5
ACLT/LG		115.0 ± 12.3	66.1 ± 15.5
ACLT/HG		114.1 ± 18.3	77.6 ± 16.8

Values represent the mean ± *SD* of seven rats per group.

a Mean value is significantly different from that of the ACLT/Cont group (*p* < .05, a Dunnett's multiple comparison test).

## 4 DISCUSSION

In this study, guava leaf extract suppressed the advancement of OA in an animal model of ACLT.

Adding guava leave extract to the diet of rats led to higher concentrations of ellagic acid in the serum and synovial membranes in a dose‐dependent manner. Ellagic acid levels in the synovial membranes inversely correlated with the cartilage destruction score. Furthermore, nuclear factor κ B activity, a transcriptional factor for ADAMTS‐5 expression (Séguin, Bojarski, Pilliar, Roughley, & Kandel, 2006), is reportedly blocked by ellagic acid (Ahad, Ganai, Mujeeb, & Siddiqui, 2014). Therefore, one mechanism by which guava leaf extract suppresses OA development may be through ellagic acid‐mediated inhibition of ADAMTS‐5 in the knee joint. Ellagic acid has been shown to have anticholestatic, antifibrotic, antihepatotoxic, and antioxidant properties (García‐Niño & Zazueta, 2015). Furthermore, ellagic acid has been reported to repress inflammation by inhibiting the expression of cyclooxygenase‐2 (COX‐2; Umesalma & Sudhandiran, 2010), an oxidative enzyme that damages cartilage (Amin, Dave, Attur, & Abramson, 2000). Thus, ellagic acid in the synovial membrane may suppress OA progression through its anti‐inflammatory action in addition to its inhibitory effects on ADAMTS‐5 activity. Further investigations are required to fully elucidate the mechanisms of ellagic acid action in the knee joint.

Guava leaf extract used in this study contained not only ellagic acid but also catechins and quercetins. Catechins and quercetins have been reported to inhibit the degradation of cartilage in vitro (Adcocks, Collin, & Buttle, 2002; Lay, Samiric, Handley, & Ilic, 2012). However, in addition to their lower inhibitory activities for ADAMTS‐5, their values in synovial membrane were too low to detect quantitatively. Thus, ellagic acid appeared to be the active ingredient in guava leaf extract that mediated the effects on knee joint destruction.

Serum COMP levels in rats from the ACLT groups increased at week 1, but not at week 10, which agrees with the findings of a previous study; Hayami et al. (2004) showed that serum COMP levels peak during the early stage of joint degradation and decline during later stages of OA. COMP levels in the ACLT/LG and ACLT/HG groups tended to be lower than those of the ACLT/Cont group at week 1; however, the effect was not significant. As the activity of ADAMTS‐5, which plays a key role in the progression of early OA (Glasson et al., 2005; Stanton et al., 2005; Verma & Dalal, 2011), was inhibited by guava leaf extract containing ellagic acid, our results indicate that guava leaf extract might be an effective therapeutic agent against early joint destruction.

## 5 CONCLUSIONS

Results from this study suggest that guava leaf extract suppresses OA development in a rat ACLT model. Inhibition of early joint destruction attributable to the action of ellagic acid in the knee joint is speculated to be an underlying mechanism of this effect. Unlike drugs that may induce adverse side effects, the safety of guava leave extract has been proven by its application in folk medicine and in the diet of various regions. However, future studies involving human subjects are needed to confirm the effectiveness of guava leaf extract in the treatment of OA.

## CONFLICT OF INTEREST

None declared.

## References

[fsn3601-bib-0001] Adcocks, C. , Collin, P. , & Buttle, D. J. (2002). Catechins from green tea (*Camellia sinensis*) inhibit bovine and human cartilage proteoglycan and type II collagen degradation in vitro. Journal of Nutrition, 132, 341–346. https://doi.org/10.1093/jn/132.3.341 11880552 10.1093/jn/132.3.341

[fsn3601-bib-0002] Ahad, A. , Ganai, A. A. , Mujeeb, M. , & Siddiqui, W. A. (2014). Ellagic acid, an NF‐κB inhibitor, ameliorates renal function in experimental diabetic nephropathy. Chemico‐Biological Interactions, 219, 64–75. https://doi.org/10.1016/j.cbi.2014.05.011 24877639 10.1016/j.cbi.2014.05.011

[fsn3601-bib-0003] Amin, A. R. , Dave, M. , Attur, M. , & Abramson, S. B. (2000). COX‐2, NO, and cartilage damage and repair. Current Rheumatology Reports, 2, 447–453. https://doi.org/10.1007/s11926-000-0019-5 11123096 10.1007/s11926-000-0019-5

[fsn3601-bib-0004] Anand, V. , Kumar, V. , Kumar, S. , & Hedina, A. (2016). Phytopharmacological overview of *Psidium guajava* Linn. Pharmacognosy Journal, 8, 314–320. https://doi.org/10.5530/pj

[fsn3601-bib-0005] Arshiya, S. (2013). The antioxidant effect of certain fruits; A review. Journal of Pharmaceutical Sciences and Research, 5, 265–268.

[fsn3601-bib-0006] Espin, J. P. , González‐Barrio, R. , Cerdá, B. , López‐Bote, C. , Rey, A. I. , & Tomás‐Barberán, F. A. (2007). Iberian pig as a model to clarify obscure points in the bioavailability and metabolism of ellagitannins in humans. Journal of Agricultural and Food Chemistry, 55, 10476–10485. https://doi.org/10.1021/jf0723864 17990850 10.1021/jf0723864

[fsn3601-bib-0007] Galois, L. , Etienne, S. , Grossin, L. , Watrin‐Pinzano, A. , Cournil‐Henrionnet, C. , Loeuille, D. , … Gillet, P. (2004). Dose–response relationship for exercise on severity of experimental osteoarthritis in rats: A pilot study. Osteoarthritis Cartilage, 12, 779–786. https://doi.org/10.1016/j.joca.2004.06.008 15450527 10.1016/j.joca.2004.06.008

[fsn3601-bib-0008] García‐Niño, W. R. , & Zazueta, C. (2015). Ellagic acid: Pharmacological activities and molecular mechanisms involved in liver protection. Pharmacological Research, 97, 84–103. https://doi.org/10.1016/j.phrs.2015.04.008 25941011 10.1016/j.phrs.2015.04.008

[fsn3601-bib-0009] Glasson, S. S. , Askew, R. , Sheppard, B. , Carito, B. , Blanchet, T. , Ma, H.‐L. , … Morris, E. A. (2005). Deletion of active ADAMTS5 prevents cartilage degradation in a murine model of osteoarthritis. Nature, 434, 644–648. https://doi.org/10.1038/nature03369 15800624 10.1038/nature03369

[fsn3601-bib-0010] Hayami, T. , Pickarski, M. , Wesolowsk, G. A. , Mclane, J. , Bone, A. , Destefano, J. , … Duong, L. T. (2004). The role of subchondral bone remodeling in osteoarthritis: Reduction of cartilage degeneration and prevention of osteophyte formation by alendronate in the rat anterior cruciate ligament transection model. Arthritis and Rheumatism, 50, 1193–1206. https://doi.org/10.1002/(ISSN)1529-0131 15077302 10.1002/art.20124

[fsn3601-bib-0011] Kamekura, S. , Hoshi, K. , Shimoaka, T. , Chung, U. , Chikuda, H. , Yamada, T. , … Kawaguchi, H. (2005). Osteoarthritis development in novel experimental mouse models induced by knee joint instability. Osteoarthritis Cartilage, 13, 632–641. https://doi.org/10.1016/j.joca.2005.03.004 15896985 10.1016/j.joca.2005.03.004

[fsn3601-bib-0012] Karai, E. , Bahlous, A. , Charni, N. , Bouzid, K. , Sahli, H. , Laadhar, L. , … Garnero, P. (2012). Association of serum levels of aggrecan ARGS, NITEGE fragments and radiologic knee osteoarthritis in Tunisian patients. Join Bone Spine, 79, 610–615. 10.1016/j.jbspin.2011.12.007 22284610

[fsn3601-bib-0013] Lay, E. , Samiric, T. , Handley, C. J. , & Ilic, M. Z. (2012). Short‐ and long‐term exposure of articular cartilage to curcumin or quercetin inhibits aggrecan loss. Journal of Nutritional Biochemistry, 23, 106–112. https://doi.org/10.1016/j.jnutbio.2010.11.004 21419610 10.1016/j.jnutbio.2010.11.004

[fsn3601-bib-0014] Lin, C.‐Y. , & Yin, M.‐C. (2012). Renal protective effects of extracts from guava fruit (*Psidium guajava* L.) in diabetic mice. Plant Foods for Human Nutrition, 67, 303–308. https://doi.org/10.1007/s11130-012-0294-0 22581156 10.1007/s11130-012-0294-0

[fsn3601-bib-0015] Mankin, H. J. , Dorfman, H. , Lippiello, L. , & Zarins, A. (1971). Biochemical and metabolic abnormalities in articular cartilage from osteo‐arthritic human hips. Correlation of morphology with biochemical and metabolic data. The Journal of Bone and Joint Surgery, 53, 523–537. https://doi.org/10.2106/00004623-197153030-00009 5580011

[fsn3601-bib-0016] Nakamura, K. (2009). Locomotive syndrome: Disability‐free life expectancy and locomotive organ health in a “super‐aged” society. Journal of Orthopaedic Science, 14, 1–2. https://doi.org/10.1007/s00776-008-1302-y 19214680 10.1007/s00776-008-1302-y PMC2779388

[fsn3601-bib-0017] Séguin, C. A. , Bojarski, M. , Pilliar, R. M. , Roughley, P. J. , & Kandel, R. A. (2006). Differential regulation of matrix degrading enzymes in a TNF alpha‐induced model of nucleus pulposus tissue degeneration. Matrix Biology, 25, 409–418. https://doi.org/10.1016/j.matbio.2006.07.002 16934445 10.1016/j.matbio.2006.07.002

[fsn3601-bib-0018] Shiozaki, M. , Maeda, K. , Miura, T. , Kotoku, M. , Yamasaki, T. , Matsuda, I. , … Inaba, T. (2011). Discovery of (1S,2R,3R)‐2,3‐dimethyl‐2‐phenyl‐1‐sulfamidocyclopropanecarboxylates: Novel and highly selective aggrecanase inhibitors. Journal of Medicinal Chemistry, 54, 2839–2863. https://doi.org/10.1021/jm101609j 21417219 10.1021/jm101609j

[fsn3601-bib-0019] Stanton, H. , Rogerson, F. M. , East, C. J. , Golub, S. B. , Lawlor, K. E. , Meeker, C. T. , … Fosang, A. J. (2005). ADAMTS5 is the major aggrecanase in mouse cartilage in vivo and in vitro. Nature, 434, 648–652. https://doi.org/10.1038/nature03417 15800625 10.1038/nature03417

[fsn3601-bib-0020] Umesalma, S. , & Sudhandiran, G. (2010). Differential inhibitory effects of the polyphenol ellagic acid on inflammatory mediators NF‐κB, iNOS, COX‐2, TNF‐a, and IL‐6 in 1,2‐dimethylhydrazine‐induced rat colon carcinogenesis. Basic & Clinical Pharmacology & Toxicology, 107, 650–655. https://doi.org/10.1111/j.1742-7843.2010.00565.x 20406206 10.1111/j.1742-7843.2010.00565.x

[fsn3601-bib-0021] Verma, P. , & Dalal, K. (2011). ADAMTS‐4 and ADAMTS‐5: Key enzymes in osteoarthritis. Journal of Cellular Biochemistry, 112, 3507–3514. https://doi.org/10.1002/jcb.23298 21815191 10.1002/jcb.23298

[fsn3601-bib-0022] Yan, C. , Lee, J. , Kong, F. , & Zhang, D. (2013). Anti‐glycated activity prediction of polysaccharides from two guava fruits using artificial neural networks. Carbohydrate Polymers, 98, 116–121. https://doi.org/10.1016/j.carbpol.2013.05.071 23987324 10.1016/j.carbpol.2013.05.071

[fsn3601-bib-0023] Yao, W. , Wasserman, Z. R. , Chao, M. , Reddy, G. , Shi, E. , Liu, R.‐Q. , … Decicco, C. P. (2001). Design and synthesis of a series of (2R)‐N4‐hydroxy‐2‐(3‐hydroxybenzyl)‐N1‐[(1S,2R)‐2‐hydroxy‐2,3‐dihydro‐1H‐inden‐1‐yl]butanediamide derivatives as potent, selective, and orally bioavailable aggrecanase inhibitors. Journal of Medicinal Chemistry, 44, 3347–3350. https://doi.org/10.1021/jm015533c 11585439 10.1021/jm015533c

[fsn3601-bib-0024] Yoshimura, N. , Muraki, S. , Oka, H. , Mabuchi, A. , En‐Yo, Y. , Yoshida, M. , … Akune, T. (2009). Prevalence of knee osteoarthritis, lumbar spondylosis, and osteoporosis in Japanese men and women: The research on osteoarthritis/osteoporosis against disability study. Journal of Bone and Mineral Metabolism, 27, 620–628. https://doi.org/10.1007/s00774-009-0080-8 19568689 10.1007/s00774-009-0080-8

